# What Is Known About Asthma Care and Management for Children and Young People Under 18 Years of age in New Zealand. A Scoping Review

**DOI:** 10.1111/crj.70139

**Published:** 2025-12-02

**Authors:** J. Blamires, M. Foster, B. Kanengoni‐Nyatara

**Affiliations:** ^1^ Auckland University of Technology, School of Clinical Sciences Auckland New Zealand; ^2^ Edith Cowan University, School of Nursing and Midwifery Perth Western Australia Australia; ^3^ AUT Child and Youth Health Research Centre Auckland New Zealand; ^4^ Migrant and Refugee Health Research Centre Auckland New Zealand

**Keywords:** adolescent, asthma, child, culturally competent care, health literacy, health services accessibility, patient care management

## Abstract

**Introduction:**

Asthma is the most common chronic health condition affecting children in Aotearoa New Zealand, with Māori and Pacific children disproportionately burdened by high morbidity and inequitable care. Despite clinical guidelines and growing research, inconsistencies in diagnosis, treatment adherence, and education persist. This scoping review identifies and maps literature on care models, service delivery, education and support strategies, and experiences of children, young people, and their family/whānau in asthma care and management for those under 18 in Aotearoa New Zealand.

**Methods:**

This scoping review was conducted according to the Preferred Reporting Items for Systematic Reviews and Meta‐analyses Extension for Scoping Reviews (PRISMA‐ScR) guidelines. We searched MEDLINE, CINAHL, Scopus, PsychINFO, and grey literature for articles published between 2014 and 2024 on asthma care for children under 18 in Aotearoa New Zealand. Eligible articles from health or community settings were thematically analysed using conventional content analysis.

**Results:**

Twenty‐one articles met inclusion criteria, including qualitative, quantitative, mixed‐methods, and policy or guideline documents. Thematic analysis revealed four interconnected themes: (1) medications and adherence, (2) education and health literacy, (3) children and whānau experiences, and (4) culture and beliefs. Findings reflect persistent inequities in asthma outcomes and care access, especially for Māori and Pacific children and highlight opportunities to strengthen culturally safe and family/whānau‐centred care to improve asthma care, treatment and its management.

**Conclusion:**

This review identifies key gaps in asthma care for children in Aotearoa New Zealand and calls for more responsive, culturally grounded models to improve asthma outcomes across diverse settings.

## Graphical abstract

Children in Aotearoa New Zealand experience variable asthma care. This review reveals critical gaps in education, treatment adherence, and culturally safe services, disproportionately affecting Māori and Pacific children. Strengthening family/whānau‐centred and culturally responsive care is vital for improving outcomes.

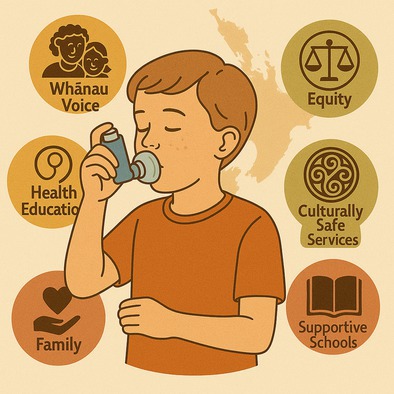

## 1 Introduction

Asthma is a complex chronic respiratory condition characterized by airway inflammation, bronchial hyperresponsiveness, and airflow obstruction [1]. Aotearoa New Zealand has one of the highest global rates of asthma symptoms and prevalence, with approximately one in five children and one in eight adults living with the condition [2].Asthma can have significant impact on day‐to‐day functioning, quality of life, school attendance, and participation in physical and social activities for children and young people under 18 years of age [3, 4, 5].

Diagnosing asthma in children is a nuanced clinical process that typically involves a combination of history taking, physical examination, and monitoring response to treatment over time [6]. When appropriately diagnosed and effectively managed, children and young people with asthma can live full and active lives, comparable to their peers without the condition [7]. The management of asthma involves a continuum of care, beginning with symptom recognition and diagnosis, followed by development of a treatment plan, often including medications and trigger management, continuing with regular follow‐up and education [8, 9]. However, inadequate or delayed diagnosis, incorrect medication use, or insufficient follow‐up may lead to poorly controlled asthma, diminished quality of life, and long‐term health complications [10].

In Aotearoa New Zealand, asthma care and management occur predominantly within primary and secondary care settings [11]. Consequently, care pathways for children and young people often cross organisations, requiring coordinated approaches The Asthma and Respiratory Foundation [ARFNZ], [12]. High‐quality asthma care must therefore include not only accurate diagnosis and effective treatment but also integration across health sectors, culturally responsive care, and tailored, ongoing education for both children and their whānau [13, 14].

Despite clear national guidelines and growing evidence about best practices in asthma care, significant disparities persist especially for Māori and Pacific children and young people [12]. Māori and Pacific children are disproportionately affected by asthma‐related morbidity and mortality, and are more likely to be underdiagnosed, undertreated, and hospitalised for asthma‐related complications [15, 16]. These inequities reflect not only the burden of disease but also systemic issues in service delivery. Māori and Pacific whānau/family frequently face barriers such as inadequate access to healthcare, a lack of culturally appropriate services, and limited opportunities for asthma education and self‐management support [17, 18, 19].

In Aotearoa New Zealand around 6 per 1000 children are admitted to hospital one or more times with a primary diagnosis of asthma each year [20]. Notably, Pacific children have the highest admission rate (12 per 1000), followed by Māori (6.6 per 1000), and then European/other children (4.3 per 1000). Younger children (aged 0–4 years) have the highest rate of admission, and boys aged 0–14 years are more likely to be admitted than girls [20]. The Environmental Health Intelligence New Zealand [21] reports that in 2022, there were 7863 hospitalisations for asthma in children aged 0–14 years, up from 6022 in 2021. This increase brought the hospitalisation rate to 818.3 per 100 000 children, the highest since 2001 EHINZ [21], and points to the need to uphold asthma as a health priority.

Children and young people under the age of 18 represent a developmentally diverse group with distinct health needs that evolve across early childhood, middle childhood, and adolescence [22, 23]. In the context of asthma, these stages involve shifting patterns of symptom recognition, diagnosis, medication management, and increasing independence in self‐care [24, 25]. Additionally, the under‐18 age group encompasses the entire period of childhood and adolescence when parental involvement in asthma management is highest and when health services, education systems, and social environments directly intersect [26].

Much of the literature on paediatric asthma in Aotearoa New Zealand aggregates data across wide age bands or prioritises either preschool‐aged children or adults, despite these stages having different symptom profiles, treatment needs, and healthcare engagement patterns [27, 28]. Further, while numerous articles have examined aspects of asthma care in Aotearoa New Zealand, including prevalence, treatment adherence, and intervention effectiveness, there has been limited synthesis of what is currently known about asthma care and management specifically for children and young people aged < 18 years. Improving asthma outcomes for children particularly for those most impacted requires a comprehensive understanding of how asthma care is delivered across settings and experienced by children, families, and health professionals. The presented review synthesises the evidence on asthma care delivery, family experiences, health education strategies, and systemic factors influencing management for children and young people with asthma. The findings will contribute to a clearer understanding of what is known, where gaps remain, and how care might be improved for this key age group.

## 2 Aim

The scoping review had the following aims:

- to identify and map the existing literature on asthma care and management for children and young people under 18 years in Aotearoa New Zealand.
- to synthesise evidence on models of care, education and health literacy, and family/whānau experiences.
- to examine cultural influences and inequities in asthma care; and

to identify gaps in the evidence and opportunities for improvement.

## 3 Methods

The scoping review was undertaken to identify and map the literature and summaries on care models, service delivery, education and support strategies, and experiences of children, young people and their family/whānau on asthma care and management of children and young people < 18 years in Aotearoa New Zealand. The review follows the five stage methodological framework developed by Arksey and O'Malley [29], with enhancements by Levac, Colquhoun and O'Brien [30] and is reported according to the Preferred Reporting Items for Systematic reviews and Meta‐Analyses extension for Scoping Reviews (PRISMA‐ScR) guidelines [31]. The research question that guided the review was “*what is known in the published literature about asthma care and management for children and young people aged under 18 years in Aotearoa New Zealand*?**”** Four databases (MEDLINE, CINAHL, Scopus, and Psych INFO) were searched twice between March and April 2025 using different key search terms commonly used in asthma research for children and parent/family populations by two authors M. F and B.K.N. See Table 1. The reference lists of articles found through the database searches were reviewed and key asthma journals were hand‐searched to identify any articles that may have been missed in the database and reference list searches. Other sources included Google Scholar, websites, and organisations.

**TABLE 1:** Search terms used.

(asthma* or acute asthma* or asthma attack or asthmatic* or asthma exacerbation or severe asthma) **AND** (adolescents or teen* or young adults or youth or children or young children or young people or tamaraki or rangatahi or family or families or parents or mother or father) **AND** (New Zealand or NZ or Aotearoa)

Aotearoa – New Zealand; rangatahi—youth; tamariki—children.

### 3.1 Study Selection

Articles were selected through establishing inclusion and exclusion criteria (Table 2). We included not only original research studies but also national guidelines, surveillance reports, programme evaluations, and relevant grey literature. This decision aligns with the purpose of the scoping review, which was to map the breadth and nature of available evidence on the topic [32]. Asthma care for children in Aotearoa New Zealand is shaped not only by peer‐reviewed research but also by policy, guideline development, and community‐based initiatives. Excluding these sources would have omitted important evidence regarding models of care, culturally responsive approaches, and service delivery contexts that are not always captured in original research articles. The programme Rayyan [33] was used to compile the articles, remove duplicates and carry out screening. The three authors, J.B; M. F and B.K.N independently performed title and abstract screening to remove articles not compatible with inclusion criteria. When the title did not provide sufficient information to determine the articles suitability, the full text was retrieved for evaluation. Full text screening was undertaken by the three authors. In cases of uncertainty or disagreement regarding an article's eligibility at any stage of screening, the three authors met to discuss and reach a collective decision. The research team together reviewed and approved the list of articles selected for full‐text review.

**TABLE 2:** Inclusion and exclusion criteria.

Inclusion criteria	Literature on child/parent/family asthma care and management of children aged < 18 years in New Zealand Published from 2014‐2025 Full text English
Exclusion criteria	Articles where data for children and young people aged < 18 years could not be extracted from the adult data Articles where the focus was on adults with asthma

### 3.2 Charting the Data and Collating, Summarising, and Reporting the Results

Relevant information from articles were extracted by lead author (J.B) into a standardized Microsoft word document that included author, title, population, age range, study design and key findings, (Table 3 **. Data Extraction Table).** We used a conventional content analysis approach to develop themes inductively from the extracted data [34]. This included thoroughly reading the findings and relevant result sections from each included study to identify recurring concepts, patterns, and issues related to asthma care and management for children under 18 years in Aotearoa New Zealand. Descriptive codes were applied to the extracted data, which were then grouped into categories The three authors reviewed and discussed these codes, refining categories through iterative comparison and reflection. These categories were further synthesised into four overarching themes: (1) medications and adherence, (2) education and health literacy, (3) children and whānau experiences, and (4) cultural beliefs. This inductive process was designed to reflect the breadth of evidence rather than to prioritise any single outcome measure. **Scope of themes**. Although clinically relevant topics such as asthma exacerbations, hospitalisations, and comorbidities were identified in several studies, they were not retained as stand‐alone themes because they were consistently reported within the broader thematic domains. For example, hospitalisations and exacerbations were discussed primarily in relation to medication adherence and systemic inequities. Comorbidities were occasionally mentioned but not as a central focus of the included literature. We therefore integrated these aspects into the thematic synthesis rather than presenting them as separate categories [34, 35].

**TABLE 3:** Data Extraction Table.

Authors	Title	Population	Age Range	Study Design	Key findings
Bush et al., 2021	Has the time come to end use of the blue inhaler?	Children and adolescents with asthma	Focus on 4–11 years and ≥ 12 years	Commentary Opinion piece	**Concerns about SABA Overuse** Overuse of short‐acting β2‐agonists (SABA, “blue inhalers”) and underuse of inhaled corticosteroids (ICS) are risk factors for asthma‐related deaths, especially in children and adolescents. **Evidence for ICS–Formoterol as Reliever** A referenced study of children aged 4–11 years found that ICS–formoterol maintenance and reliever therapy: Reduced risk of severe asthma attacks by two‐thirds compared to conventional therapy. showed greater efficacy than in adolescents and adults, highlighting its promise for younger children. **Call for Policy Shift** Authors argue that continuing to prescribe SABA monotherapy, especially in those aged 12 and older, is unjustified and should be replaced with ICS–formoterol. ‐urge for more research to determine whether a similar shift should be made for children under 12. **Aotearoa New Zealand Context** References NZ's historical action in withdrawing fenoterol, which led to a > 50% reduction in asthma mortality within a year—a precedent for bold regulatory change.
Chan et al., 2016	Factors associated with medication adherence in school‐aged children with asthma	Children aged 6–15 years (*n* = 101) with asthma, recruited from an Auckland ED after exacerbation, on ICS. All had been prescribed twice‐daily inhaled corticosteroids. Participants were drawn from the control group of an RCT (Chan 2015)	6–15 years	Observational analysis (adherence predictors)	**Overall Adherence Was Poor** Median adherence was 30% of prescribed doses (IQR 17–48%). **Four Factors Significantly Associated with Better Adherence** Identified via multivariable regression: Female sex (+12% adherence) Asian ethnicity (+19%) smaller household size (−3% per person added) Younger age at diagnosis (+2.7% per year earlier) Together, these four factors explained 30% of the variation in adherence (adjusted R^2^ = 0.30). **No Association Found with Learning Style** Aural learning preference had no impact on adherence. **Other Factors Not Statistically Significant** Socioeconomic deprivation‐Parental education‐Asthma knowledge or responsibility score‐Access to doctors/pharmacies‐Delayed healthcare or medication pick‐up due to cost **Ethnic Differences in Adherence Patterns** Asian children: 47% adherence Māori: 19% adherence NZ European: 26% adherence Pacific peoples: 28% adherence Māori children had the poorest adherence, followed closely by NZ European and Pacific peoples. IN SUMMARY: female sex, Asian ethnicity, smaller household size and younger age at diagnosis were strongly associated with better preventive medication adherence in children with poorly controlled asthma
Chan et al., 2015	The effect of an electronic monitoring device with audiovisual reminder function on adherence to inhaled corticosteroids and school attendance in children with asthma: a randomised controlled trial	Children aged 6–15 years (*n* = 220) with physician‐diagnosed asthma, recently treated in an Auckland ED, on inhaled corticosteroids. Participants were randomly assigned to intervention (EMD + audiovisual reminder) or control (EMD only).	6–15 years	Randomised Control Trial (RCT)	**Adherence to Preventive Medication** Adherence was significantly higher in the intervention group: Median adherence: 84% (intervention) vs. 30% (control) 70% adherence achieved by more children in the intervention group. **Asthma Control Improved** Asthma morbidity score significantly reduced in the intervention group: Mean reduction: 2.0 points vs. 1.2 points in control (*p* = 0.008) Childhood Asthma Control Test scores were significantly better at 2, 4, and 6 months (*p* < 0.0001). **Reliever Use Lower in Intervention Group** Median days of reliever use: 9.5% vs. 17.4% (*p* = 0.002) **Fewer Asthma Exacerbations (Initial Period)** In the first 2 months: 6% of intervention group vs. 24% of control group had ≥ 1 exacerbation (*p* = 0.015). This difference was not sustained at 4 and 6 months. **No Significant Change in School Absences** Absence from school was similar between groups: 1.9% of days (intervention) vs. 1.7% (control) — *p* = 0.99
Chan et al., 2023	Asthma exacerbations in New Zealand 2010–2019: A national population‐based study	Patients with asthma were grouped into five age categories (*< 5*, 5–14, 15–44, 45–64, and ≥ 65 years) with patient age calculated at 1 January of each year.	5–14 years	Retrospective population‐based observational cohort study covering the ten years 2010–2019	In 2010 and 2019, asthma **prevalence was** higher for children under the age of five years than in other age groups The age group 5–14 years had a prevalence of 15.9% in 2010 and 15.4% in 2019, which is the second‐highest prevalence after the under‐5 group in 2010 **Exacerbation rates** increased in all age groups except for children under five years or the **5–14‐year** age group, exacerbation rates increased from: **305.5 per 1000 patient‐years (2010)** to **365.8 per 1000 patient‐years (2019)** **Adjusted IRR** = **1.189** (95% CI: 1.168–1.210), *p* < 0.001 Hospitalisation rates for 5–14 years: 2010–24.3 admissions per 1000 patient‐years 2019–19.1 admissions per 1000 patient‐years Adjusted IRR = 0.813, showing a significant reduction in hospitalisations for this group (p < 0.001)
Eathorne et al., 2025	Reducing ethnic inequities: Patterns of asthma medication use and hospital discharges in Māori in Aotearoa New Zealand.	National dispensing & hospitalisation data from Māori and non‐Māori children in Aotearoa New Zealand. Data span: 2013–2023, including COVID‐impacted years.	Aged 12 + but dedicated section on > 12 years		**Dispensing an medication use**. Budesonide/formoterol use increased only modestly in under‐12 s from a very low baseline. This limited uptake is attributed to the lack of clinical trial evidence and paediatric guidelines not recommending ICS/formoterol as a preferred reliever in this age group. **SABA Use** Māori children had ~50% higher SABA dispensing rates than non‐Māori throughout the study period. SABA dispensing decreased until 2021, then increased again for both Māori and non‐Māori. **Hospitalisations** Asthma hospital discharge rates for Māori were consistently higher than for non‐Māori across the 10‐year period. Hospitalisation rates for children decreased until 2021, then increased again, especially for Māori. This contrasts with older age groups (12+) where hospitalisations declined. **Key Messages for Asthma Care (Children < 12 Years)** High reliance on SABAs, particularly among Māori tamariki, persists. Underuse of ICS/formoterol regimens due to age‐based prescribing guidelines. Māori children remain disproportionately affected by asthma hospitalisations. Recent increase in hospital admissions (post‐COVID) suggests a renewed need for tailored interventions. Emphasises the need for paediatric‐specific strategies to reduce inequities—distinct from adult/adolescent care models.
Hatter et al., 2021	The Children's Anti‐inflammatory Reliever (CARE) study: a protocol for a randomised controlled trial of budesonide‐formoterol as sole reliever therapy in children with mild asthma	A total of 380 participants aged 5 to 15 years with asthma (diagnosed by any doctor), only using a SABA reliever	5–15 years	Protocol for an RCT	Eligible participants will be randomised 1:1 to receive as needed for relief of asthma symptoms: 1. Intervention: Budesonide‐formoterol 50/3 μg pressurised metered‐dose inhaler (Symbicort Rapihaler, AstraZeneca) two actuations via spacer (Space Chamber Plus, Medical Developments International ltd., Victoria, Australia). 2. Control: Salbutamol 100 μg pressurised metered‐dose inhaler (Ventolin, GlaxoSmithKline, Brentford, UK) two actuations via spacer (Space Chamber Plus, Medical Developments International Ltd).
Hebenton, 2015	Exploring the asthma interventions of rural pharmacies. Pharmacists' experiences and the HAPA model.	Caregivers and pharmacists involved in managing asthma in children. Children with asthma were referenced but not directly studied.	While specific ages of the children are not always given, it is clear that the focus is on school‐aged children, imply an age range of 5–12 years	Qualitative exploratory study	**Parental responsibility** is critical for effective asthma management. Parents are typically the key decision‐makers, though some may overestimate children's ability to self‐manage. Communication breakdowns in blended or separated families negatively affect adherence—e.g., inhalers getting left behind or not transferred between homes. P**harmacist**s play an active role in supporting families, including issuing duplicate inhalers for multiple homes. Children's developmental stage influences how involved they can be in self‐management. Some caregivers delegate too early; others struggle to let go as children mature. Pharmacist perspectives reveal gaps in parental understanding and highlight the importance of family dynamics and routines in maintaining adherence. **Social and structural barriers** (e.g., time, single parenting, childcare logistics) affect engagement in asthma education and care.
Jayamini et al.,2022	State of Asthma‐Related Hospital Admissions in New Zealand and Predicting Length of Stay Using Machine Learning	Children and adults admitted to hospitals in Auckland DHB's (2017–2021)	Data includes children under 18 (specific mention of under 10 year olds having distinct admission patterns)	Data analysis and machine learning modelling	Most asthma admissions were in children Length of stay (LOS) shorter in younger children (Children under 10 years had shorted average hospital stays then older age groups) Māori children had longer average LOS‐ Māori females had the longest average LOS among all groups (2.8 days) Highest asthma related hospital admissions occurred June–August (seasonal triggers) Machine learning model was trained to predict LOS using age, primary diagnosis, ethnicity, DHB and performed well Random Forest algorithm achieved a mean squared error (MSE) of 0.43, which was lower than the MSEs for the other models they tested (Decision Tree and Gradient Boosted Tree)
Jones et al., 2015	He Māramatanga Huangō: Asthma Health Literacy for Māori Children in New Zealand	Māori children with asthma aged 4–18 years (*n* = 20), and their parents/caregivers, in Wellington and Auckland regions. Study also included input from:800 + health professionals across Aotearoa New Zealand (including nurses, GPs, pharmacists, and asthma educators); 35 in‐depth interviews with providers from Māori‐led and mainstream services in Auckland and Wellington.	Aged 4–18 years	Mixed methods (survey, interviews, resource review) Report Kaupapa Māori Research (KMR) methodology	**Whanau/Family** **Significant Health Literacy Barriers** Fewer than half of whānau were confident in understanding asthma or using asthma plans. Parents could often do asthma tasks (e.g., inhaler technique) but did not understand why those tasks were important. Lack of action plans and limited understanding of symptoms, severity, and medications were common. **Structural Barriers in Care Delivery** Health professionals reported difficulty assessing families' understanding and often defaulted to verbal‐only education. System‐level constraints (short consultations, lack of follow‐up) made consistent asthma education difficult. **Disconnection Between Whānau and Providers** Whānau reported feeling: Not listened to or taken seriously Judged or ashamed when asking questions Frustrated by inconsistent messages and seeing different providers each visit **Preference for Whānau‐Centred, Culturally Safe Education** Parents valued: Trusted relationships with a regular GP Visual, hands‐on, and stepwise asthma education Māori‐specific, culturally relevant resources and approaches **Medication Patterns and Disease Severity** Many children (ages 4–18) had moderate‐to‐severe asthma: 60% had been hospitalised at some point 65% had been prescribed oral corticosteroids Only 55% had an asthma action plan Most common triggers: colds/flu, weather, dust, exercise **From clinicians** *Difficulty Assessing Asthma Literacy* Many clinicians admitted they struggled to judge whānau understanding of asthma. often relied on verbal cues or assumed understanding, rather than confirming it explicitly. Some used body language or tone to estimate comprehension, which was acknowledged as unreliable. *it's hard to tell how much they really understand… sometimes you can tell they are just nodding*. — Nurse (p. 21) **Time Pressures and Fragmented Care** Short consultation times and lack of continuity meant asthma education was often superficial or incomplete. Health professionals rarely had time to explain preventer medication properly or check inhaler technique. *I do not think I've ever had enough time to really go through it the way I'd like to*. — GP (p. 22) **Verbal‐Only Communication is Common but Limiting** Many clinicians relied primarily on verbal explanations, with limited or no use of written materials, action plans, or hands‐on demonstration. Some acknowledged that verbal‐only education is inadequate, especially when addressing health literacy challenges. We do all this talking, but no one checks if it's sinking in. — Pharmacist (p. 23) **Lack of Cultural Confidence and Culturally Tailored Tools** Many providers wanted more training in delivering asthma care in culturally appropriate ways. Some felt uncertain about how to adapt communication styles or resources for Māori whānau. *It's not just about language—it's about knowing how to build trust and meet people where they are at*. — Community health nurse (p. 24) **Desire for Whānau‐Centred Models** Providers recognised the value of long‐term relationships and holistic, family‐centred approaches. Several acknowledged that Māori‐led services were more successful at engaging whānau effectively. They [kaupapa Māori services] do it better—because they take time, and it's not just medical— GP (p. 25)
Jones et al., 2016	Self‐efficacy in managing chronic respiratory disease: Parents' experiences	Parents (*n* = 23) of children aged 0–16 years with chronic respiratory disease (including asthma), hospitalised in a NZ tertiary hospital. Subgroup: 10 children with asthma Participants included both mothers and fathers, though most primary caregivers were mothers.	Most children were aged 3–6 years (*n* = 10), but full age range was 0–16 years	A **qualitative** study using general inductive thematic analysis. Semi‐structured interviews with 23 parents whose child (0–16 yrs) was in hospital.	**Self‐efficacy shaped by a sense of overwhelming responsibility** Primary caregivers (often mothers) experienced an intense, ongoing sense of accountability for managing the child's condition. This burden impacted their confidence and well‐being, especially during acute events or hospitalisation. *It's just not like you can put that responsibility onto someone else*. — (A15) **Lack of shared responsibility within families** Fathers/secondary caregivers often lacked confidence to contribute fully, citing lack of experience or fear. As a result, mothers often became the “sole knower” of the child's condition and treatments. *I do not want to be the sole ‘knower’ of it all, but it's the way it's worked out. — (A13)* **Relationships with health professionals affect confidence** Supportive, consistent relationships with primary care teams (especially respiratory nurses and GPs) enhanced parents' self‐efficacy. Conversely, lack of continuity, pressure in hospital settings, and feeling dismissed reduced parental confidence. **Unpredictability of asthma exacerbations increased anxiety** Sudden onset of symptoms (within 2 h) contributed to constant vigilance and emotional stress. Parents described the burden of having to judge whether to treat at home or escalate care. *it's really, really stressful … it takes up a huge amount of time and energy* **Family dynamics and role division are affected** Siblings were often impacted emotionally or socially due to the focus on the child with respiratory disease. Caregiving responsibilities strained work and household routines.
McNamara et al., 2020	Asthma And Respiratory Foundation New Zealand Child Asthma Guidelines: a quick reference guide	Children aged 0–11 years The guidelines apply to all children aged ≤11 years Adolescents (12+) are covered in a separate guideline	Aged 0–10 years	Clinical guideline (evidence‐based consensus)	**Guideline‐Based Asthma Management** Diagnosis based on clinical symptoms and response to therapy Emphasises trial treatment and regular review in 5–11 age group Spirometry recommended for children 6 + years **Clear Stepwise Treatment Pathway (5–11 years)** Includes five steps of increasing intensity, from no maintenance to oral steroids and biologics ICS is mainstay for regular symptom management Emphasis on device appropriateness by age and use of spacer with pMDI **Strong Equity Focus** Māori and Pacific children are: More likely to have severe symptoms and hospitalisation Less likely to receive ICS, action plans, and adequate education **Guidelines recommend:** Cultural responsiveness Referrals to Māori/Pacific health providers Use of multilingual resources **Importance of Health Literacy and Education** Education should be: Developmentally appropriate and repeated over time in small “chunks” Use simple language and pictorial tools (e.g., www.pamp.co.nz) “Use the term ‘asthma flare‐up’ instead of ‘asthma exacerbation’, and ‘puffer’ instead of ‘inhaler’.” **Asthma Action Plans for Every Child** Must be: Individualised and culturally appropriate Shared with schools, childcare, and multiple caregivers Reviewed annually or more frequently as needed **Non‐Pharmacological Recommendations** Address: Cold/damp housing Tobacco exposure Barriers to access Encourage: Physical activity Influenza vaccination from 6 months of age
Pattemore et al., 2020	Changes in asthma severity in the first year of school and difficulty learning to read	Children aged 5 years entering primary school in New Zealand; subgroup with asthma (*n* = 55) and control group without respiratory symptoms (*n* = 74) Participants were part of a cohort study across 8 randomly selected NZ schools. Asthma group diagnosed via parent report + ISAAC criteria. Reading performance was measured at school entry (T1) and one year later (T2).	5–6 years	Observational cohort study	**Asthma and Early Reading Achievement** Children with asthma at school entry were significantly more likely than controls to be in the lowest quartile for reading after one year. This association was not explained by school absence, life events, behaviour problems, or parent mental health. **Persistence of Asthma Symptoms Matters** Within the asthma group, children with persistent symptoms (versus improved symptoms) had: 2 × greater odds of being in the lowest quartile for reading. Significantly lower reading age scores in both word and story reading at the end of year one. **Predictors of Low Reading** In regression models, only two predictors were retained: Word reading score at school entry Persistence of asthma symptoms Persistent asthma had a stronger association with low reading than reading readiness at entry. **No Difference in Attendance or Other Confounders** Children with asthma did not miss significantly more school than controls. No significant differences in life stress, behaviour problems, or parental mental health between groups.
Schlichting et al., 2021	Childhood asthma in New Zealand: the impact of on‐going socioeconomic disadvantage (2010–2019)	All children aged 0–14 years in Aotearoa New Zealand (*n* = 39 731 asthma hospitalisations; 5.5 million prescriptions). Data were collected from public hospital admissions and pharmaceutical claims from 2010 to 2019. asthma as a discharge	0–4 years 5–9 years 10–14 years	Observational study (Retrospective analysis of public hospital admission and pharmaceutical prescription data)	Decline in Hospitalisations, but persisting Inequities Asthma hospitalisations decreased by: 62% in 0–4‐year‐olds, 21% in 5–9‐year‐olds, 3% in 10–14‐year‐olds Māori children were hospitalised at more than double the rate of non‐Māori (7.2 vs. 3.5 per 1000) **Socioeconomic Disparities Are Persistent** Children from the most deprived areas were: Hospitalised at 2.8 × the rate of those from least deprived areas showed greater seasonal variation in admissions, especially during winter These disparities remained unchanged over the 10‐year period. **Preventer vs Reliever Prescription Patterns** Reliever medications (e.g., salbutamol) accounted for 50% of asthma prescriptions Māori children in the most deprived areas had: Higher reliever prescription rates (377/1000) Lower preventer prescription rates than non‐Māori indicates greater reliance on acute care and lower preventive management for Māori tamariki. **Readmission and Length of Stay Inequities** Māori children: Were more likely to be readmitted within 90 days (18% vs. 14% for non‐Māori) Had longer hospital stays than non‐Māori Cost Burden Estimated total direct cost of paediatric asthma (2010–2019): $165 million $103 M = hospitalisations, $62 M = prescriptions, Māori children had twice the hospitalisation cost per capita compared to non‐Māori.
Sudarsan et al., 2023	Navigating asthma—the immigrant child in a tug‐of‐war: A constructivist grounded theory	Indian immigrant children with asthma (*n* = 9) and their family carers (*n* = 10), living in Aotearoa New Zealand.	Children aged 8–17 years (With several children aged 8–12, including 8, 9, 10, and 12‐year‐olds)	**Qualitative Design** Constructivist grounded theory design.	The theory, *navigating asthma: the immigrant child in a tug‐of‐* *war*, is the resulting grounded theory with the *tug‐of‐ war* being the basic social process. This theory describes Indian immigrant children as caught in two tensions: 1.Between Indian and New Zealand cultural expectations 2.Between their own preferences and those of their caregivers **Being Fearful** ⦁Fear of symptoms, stigma, blame, and treatment side effects ⦁Children expressed anxiety about attacks, inhaler use, and exclusion from sports ⦁Family beliefs about cold foods, milk, and outdoor activity influenced child restrictions Do not *eat ice creams. Do not play outside … only on sunny days* (8‐year‐old participant) **Seeking Support** ⦁Children were influenced by parents, peers, and extended family ⦁Misinformation and cultural stigma were passed down from caregivers ⦁Children often wanted to follow medical advice but were overridden by caregivers *Mum keeps the inhaler. She does not want me to use it (12‐year‐old participant)* **Clashing Cultures** ⦁Differences between Western biomedical advice and traditional Indian beliefs ⦁Parents controlled access to treatment (e.g., restricting inhalers) based on cultural concerns ⦁Children desired more autonomy, especially as they grew older *Doctors say use it [preventer], but mum says it's not good (8‐year‐old participant)* **Additional Themes:** ⦁Authoritarian parenting style: limited children's involvement in their care ⦁Stigma: parents avoided disclosure of asthma to schools, relatives, or marriage prospects ⦁Acculturation stress: parents struggled with NZ health system, lack of extended family, and financial/work pressures
Telfar‐Barnard & Zhang, 2021	The impact of respiratory disease in New Zealand: 2020 update	Respiratory disease indicators including asthma	Age group covered: Primarily data are grouped as 5–9 years and 10–14 years, with some combined analysis of 5–14 years.	Descriptive national surveillance report	Asthma prevalence in children (2019/2020): 5–9 years: 17.5% 10–14 years: 13.6% Māori children had the highest prevalence: 22.4% Asian children had the lowest prevalence: 7.6% Prevalence increased with socioeconomic deprivation **Hospitalisation:** Asthma hospitalisation rates for children aged 5–14 years were lower than for those under 5, but disparities remain. Māori and Pacific children experienced higher hospitalisation rates and readmission within 90 days compared to non‐Māori/non‐Pacific children **Health inequities:** Children living in high deprivation areas had significantly higher asthma admission rates—up to 2.8 times more than those in the least deprived areas. Seasonal spikes in admissions were more pronounced for children from the most deprived quintiles **Medicated asthma trends (2006–2020):** Rates have remained relatively stable over time. Boys consistently had higher rates than girls. No significant overall trend change noted in this age range over time, but ethnic and deprivation‐based disparities persisted **Guideline changes impact diagnosis:** Since 2012, children under 5 are now less likely to be diagnosed with asthma due to changes in diagnostic practices. This affects prevalence comparisons, especially for the 5–9 group transitioning from undiagnosed preschool wheeze
The Asthma and Respiratory Foundation NZ, 2018	Lessons learned from the ARFNZ Partnership Pilot with Turuki Health Care: Combining school‐based screening with in‐home assessments to improve asthma diagnosis and management	Māori and Pasifika children aged 5–12 living in high deprivation areas of South Auckland (Māngere), and their whānau. Schools involved: Te Kura Kaupapa Māori ō Waatea, Ngā Iwi Primary, Southern Cross Campus, and Te Kura Kaupapa Māori ō Māngere Programme targeted underdiagnosed and poorly managed asthma among tamariki	5–12 years	Pilot programme evaluation (mixed methods)	**Screening and Assessment** 994 children screened (79% of total school roll) 122 initial in‐home assessments completed 21 children received completed **Child Asthma Action Plans** 59 referrals made to Respiratory Nurse Specialists **Barriers to Care** Low trust in health services High transience and unstable contact information Fatigue from multiple service engagements (“intervention fatigue”) Cost and time barriers despite free GP care for children Cultural beliefs (e.g., asthma as a weakness, reluctance to use preventers) *Some members of his whānau were initially opposed to the use of a preventer … believing it could lead to weakness (p. 22)* **Value of In‐home Education** Asthma Nurse built trust over time through **multiple home visits** Whānau received tailored asthma education Many learned for the first time that the child's symptoms were **not normal or safe** *Education has been the key in this case—educating in small amounts so that it's not overwhelming (p. 22)* **School and Community‐Based Interventions work** Shift from home‐distributed questionnaires to **school‐based screening** increased response rates Leveraging existing Mana Kidz programme improved engagement School staff and School‐Based Health Teams received asthma training **Environmental Health Factors** Many homes assessed were cold, damp, mouldy Referrals made to AWHI (Auckland Wide Healthy Homes Initiative)
The Asthma and Respiratory Foundation NZ, 2023	Asthma and Respiratory Foundation NZ (ARFNZ) Survey 2023	Adults with asthma and parents of children with asthma (*n* = 488 total; includes respondents aged 6–12 years) Although the survey wasn't exclusive to the 5–12 age group, it included children aged 6–12, with insights provided by parents.	41 children aged ≤12 years who required emergency care in the past year. Clear subgroup of respondents representing children aged 6–12 years.	National cross‐sectional survey (mixed methods)	**Asthma Control** 64% of children aged 12 and under had poorly controlled asthma based on guideline criteria. Many caregivers overestimated their child's asthma control (74% believed it was well‐ or mostly controlled). **Access to Care** 65% of parents were concerned about getting an appointment when needed. 33% of parents reported asthma significantly reduced their child's quality of life. *My child has not been assigned a regular GP– she just sees whoever is available at the practice—this has been a huge barrier for us in regard to continuity of care*. **Asthma Reviews and Management** Only 57% of children had an asthma action plan. 46% of children who visited ED/After Hours received follow‐up within a week, per guidelines. **Medication and Technique** Spacer use: While recommended for all children using MDIs, many still reported not always using one; stigma and practicality were barriers. Reliever overuse: Many children were using salbutamol inhalers more than 3 times/year, contrary to best‐practice guidelines. *There is very limited education for children in schools to reduce the stigma of using inhalers/devices which therefore greatly reduces the likelihood of a child using it*. **Education and Support** Families wanted better asthma education in schools and for teachers to recognise subtle signs of asthma attacks. Some quotes reflected difficulty accessing a regular GP or feeling unheard by healthcare professionals. *I would like teachers in schools to be made aware of the subtle signs of an asthma attack in the classroom*
Tibble et al., 2019	Heterogeneity in Asthma Medication Adherence Measurement	Children aged 6–15 years (*n* = 211), all with physician‐diagnosed asthma and recently treated in an Auckland ED for an asthma attack. Participants were on twice‐daily inhaled corticosteroids (ICS). This was a secondary analysis of a previous NZ‐based RCT using electronic monitoring devices (EMDs).	Aged 6–15 years	Quantitative experimental study. Secondary analysis of RCT	**Medication Adherence is Highly Variable** Substantial heterogeneity in adherence behaviours across participants. Single aggregate adherence measures (e.g., % of doses taken) failed to detect meaningful differences in medication‐taking patterns. **Impact of Reminder‐Based Interventions** Children in the intervention group (EMDs with audio‐visual reminders): Skipped both daily doses on only 3% of days, vs. 49% in the control group. 35% of children took both doses on > 80% of days (vs < 2% in control). Had fewer and shorter treatment intermissions (pauses in medication). **Adherence Declines Over Time** The time delay between the reminder tone and dose‐taking increased across the study (from ~3 min to ~53 min). Fewer children maintained full adherence over time, suggesting challenges with long‐term implementation. **Patterns of Non‐Adherence identified** Some children: Skipped both doses on non‐consecutive days Had long gaps of no medication (treatment intermissions) Took only one daily dose (often missing mornings)
Tomlin et al., 2018	Trends in Outpatient Prescription Medicine Use in New Zealand Children 2010–2015: A National Population‐Based Study	Children registered with a NZ general practice from 2010‐2015	Children aged<18 years	Retrospective observational	While this study focused on lots of different medications‐ data extracted only related to asthma related medication trends Salbutamol (SABA) was the most widely prescribed respiratory drug—dispensed to ~12% of all children annually Inhaled corticosteroid (ICS) use decreased by 17.5% from 2010‐2015 (Includes fluticasone, beclomethasone, and budesonide) Use of combined ICS/LABA (e.g., fluticasone + salmeterol) increased significantly Oral corticosteroids (prednisone, prednisolone) use was relatively stable in older age groups, higher in children < 6 **High variation in medicine use by ethnicity:** Pacific and Asian children had highest prescription rates Māori children had lower prescription rates than Pacific and Asian children but slightly higher than NZ European children **Summary key relevant findings**. Potential underuse of preventer medication (ICS) among children Over‐reliance on reliever medications (SABAs) Medication patterns raise questions about adherence to clinical guidelines and equity in prescribing
Trnka, 2014	Domestic Experiments: Familial Regimes of Coping with Childhood Asthma in New Zealand	Parents (primarily mothers) of children with asthma (*n* = 17), plus health professionals and young adults with childhood asthma in NZ (Auckland, Wellington, Dunedin)	Not always specified	Qualitative ethnographic Semi structured interviews	**Parent‐Experts and Familial Regimes of Care** Parents—especially mothers—positioned themselves as experts on their child's asthma, sometimes overriding or modifying medical advice. Families conducted “domestic experiments”, developing personalised, experience‐based regimes to manage asthma at home. **Tension Between Compliance and Autonomy** While clinical discourse framed self‐management as compliance with guidelines, parents often redefined care routines to suit their child's triggers, behaviours, and family norms. Use of preventers and relievers was often adaptive and situational, rather than strictly guideline driven. **Mothers' Central Role in Asthma Management** Mothers were most often the primary decision‐makers, care coordinators, and interpreters of symptoms. Care was described as gendered—fathers provided support but rarely led care planning. **Pharmaceutical Use and ‘Medico‐Normality’** Two strategies were identified: Daily adherence to preventative medication, aimed at controlling symptoms Intermittent reliever use, maintaining asthma as an episodic condition Both approaches sought to preserve the idea of a ‘normal childhood’. **Child Involvement in Care** Some parents encouraged young children to begin recognising symptoms and initiating treatment. True autonomy in care was rarely granted until late adolescence.
Wiechern, 2014	Analysis of breathing during oral reading by young children with and without asthma using non‐contact respiratory monitoring methods: a preliminary study of task and reading difficulty effect	Children aged 5–9 years (*n* = 22), including 11 with moderate‐to‐severe asthma and 11 age‐ and gender‐matched controls without asthma, in New Zealand.	5–9 years	Quantitative observational study (thesis)	Children with asthma adjust breathing while reading aloud 82% of children with asthma breathed more slowly when reading more difficult texts. These children also paused longer and had longer expirations during reading. Breathing adjustments were negatively associated with asthma severity (*p* = 0.046). Reading aloud physiologically more demanding for children with asthma Asthma may affect oral reading fluency, particularly under cognitive load (i.e., reading difficult texts). The study found significant differences in pause time and expiration time between children with and without asthma during reading tasks. Breathing disruptions may contribute to reduced reading fluency, especially during foundational school years.

Abbreviations: ARFNZ, Asthma and Respiratory Foundation New Zealand; AWHI, Auckland Wide Healthy Homes Initiative; CI, Confidence Interval; DHB, District Health Board; ED, Emergency Department; EMD, Electronic Monitoring Device; GP, General Practitioner; HAPA, Health Action Process Approach; ICS, Inhaled Corticosteroids; ICS–Formoterol, Inhaled Corticosteroid and Formoterol; IQR, Interquartile Range; IRR, Incidence Rate Ratio; ISAAC, (International Study of Asthma and Allergies in Childhood; KMR, Kaupapa Māori Research), Kura Kaupapa Māori, Māori‐medium school; LABA, Long‐Acting Beta₂‐Agonist; Mana Kidz, school‐based child health programme; MDI, Metered‐Dose Inhaler; MSE, Mean Squared Error; pMDI, Pressurised Metered‐Dose Inhaler; RCT, Randomised Controlled Trial; SABA, Short‐Acting Beta₂‐Agonist; tamariki, children; whānau, family; adjusted R^2^, Adjusted Coefficient of Determination; Kaupapa Māori ‐Māori, a philosophical doctrine, incorporating the knowledge, skills, attitudes and values of Māori society.

## 4 Results

**FIGURE 1:**
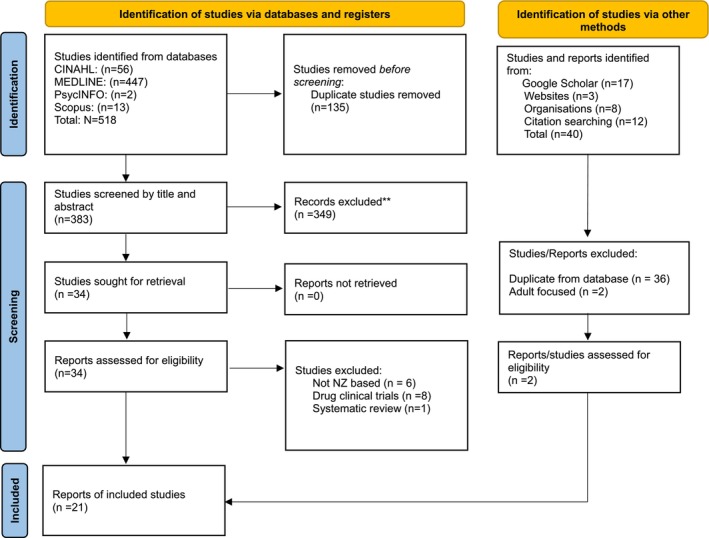
Preferred Reporting Items for Systematic Reviews Flowchart Identification of studies via other methods.

A total of 518 records were identified through database searches (CINAHL, MEDLINE, PsycINFO, Scopus). After removing 135 duplicates, 383 records were screened by title and abstract where 349 were excluded. Of the 34 full‐text articles assessed for eligibility, 15 were excluded (6 not Aotearoa New Zealand based, 8 clinical drug trials, 1 systematic review). An additional 40 records were identified via other sources (Google Scholar, websites, organisations, citation searching), of which 38 were excluded (−36 duplicates and 2 adult‐focused studies). This process resulted in 21 articles being included in the final review. (Figure 1).

**FIGURE 2:**
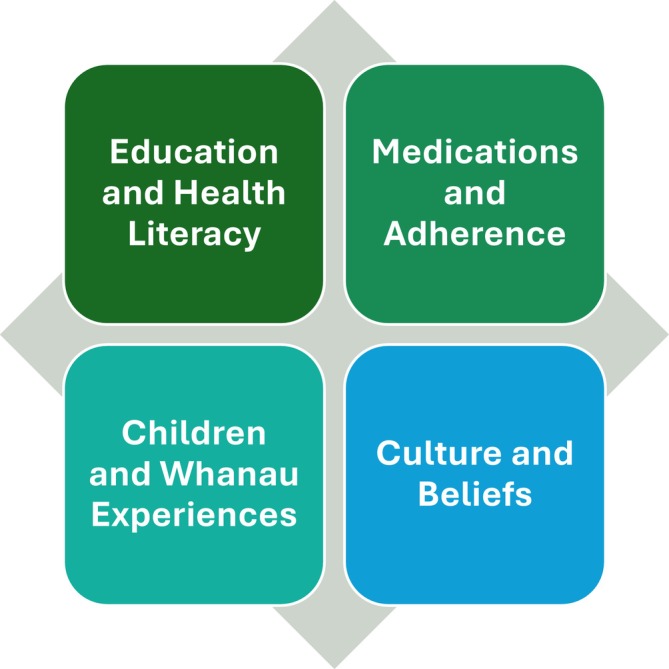
Asthma care, management and treatment in Aotearoa New Zealand key themes.

The 21 articles encompassed 11 quantitative articles, four qualitative articles, four mixed‐methods, and two evidence‐based policy or guidelines. Thematic analysis of the articles generated four themes, being medications and adherence; education and health literacy; culture and beliefs; and children and whanau/family experiences; (Figure 2. Asthma care, management and treatment in Aotearoa New Zealand key themes).

### 4.1 Medications and Adherence

Increased medication adherence leads to better asthma control and health outcomes. However, many articles found that patient adherence to medication is poor, in children further revealing important insights into factors that influence adherence and the potential for improved management strategies [14, 36, 37, 38, 39, 40]. Chan et al. [37], identified low adherence rates (median adherence of 30%) to asthma medication, with notable differences linked to size of household, gender and ethnicity, particularly among Māori children who exhibited the poorest adherence. This study along with their previous work [36] demonstrated an electronic monitoring device with an in‐built audiovisual reminder was beneficial in tracking and improving adherence. Tibble et al. [38] also found that some children had long gaps in medication use, while others frequently skipped doses or took irregular breaks, leading to poor adherence patterns. Jones et al. [14] noted that general practitioners recognized families may have misconceptions or lack adequate knowledge about asthma, impacting adherence. General practitioners therefore often focused on medication compliance during asthma education as also reported by Trnka [39]. Trnka [39] further challenges the traditional view of noncompliance, suggesting that many families were actively engaged in alternative forms of self‐care, which they believed were in the best interest of their child. Families reported using creative strategies to integrate asthma management into daily life, including medication adjustments and tailored routines. Trnka [39] described these as ‘*domestic experiments*,’ where families personalised treatment plans based on experience, intuition, and observation. For example, some were committed to daily preventive medications, some chose to use homeopathic medicine to reduce the need for inhaled steroids and others admitted refraining from daily preventors relying solely on SABA inhalers. This study highlights the need for a more nuanced understanding of adherence behaviours and a more collaborative approach to asthma management [39].

The overuse of short‐acting β2‐agonists (SABAs) and underuse of inhaled corticosteroids (ICS) as well‐established risk factors for asthma‐related morbidity and mortality in children and adolescents was highlighted in several studies [12, 41, 42, 43, 44]. Importantly, Māori children were noted to have persistently higher dispensing rates of SABAs compared to non‐Māori, reflecting systemic inequities in asthma treatment access and practice [42, 43]. Drawing attention to this issue of overuse of SABA's, Bush et al. [44] highlighted how the Global Initiative for Asthma's recommendation of inhaled corticosteroid, being formoterol as the preferred reliever over SABAs. Formoterol was shown to significantly reduce severe asthma attacks in patients over 12 years old. They further recommended for regulatory bodies to mandate SABA use as off‐label for this group of children with further research required. In a similar vein, ARFNZ survey [12] noted Salbutamol reliever inhalers were overprescribed, overused, and lacked regular asthma reviews, therefore putting children at risk. Hatter et al. [41] further addressed the issue of overprescription of SABA with their proposed intervention study aimed to determine the efficacy and safety of as‐needed budesonide‐formoterol therapy compared with as‐needed salbutamol in children aged 5 to 15 years with mild asthma, who only use a SABA.

The ARFNZ Asthma Guidelines: a quick reference guide provides evidence based guidelines for approaches to treatment with asthma medications including a stepwise approach to the introduction of inhaled corticosteroids for children under 12 years of age [45]. Despite evidence‐based recommendations and Māori children being hospitalized for asthma at twice the rate of non‐Māori (7.2/1000 vs. 3.5/1000, *p* < 0.001) with higher 90‐day readmission rates (18% vs. 14%, p < 0.001), they consistently received a lower proportion of corticosteroid prescriptions for asthma [46].

### 4.2 Education and Health Literacy

Five articles focused on asthma health education and/or health literacy from the perspective of family members, and health care professionals in terms of recommendations for practice [12, 14, 45, 47, 48]. Jones et al. [14] reported whānau often felt under‐informed on the importance behind asthma action plans including asthma triggers, understanding severity and disease stages and practical knowledge like using an inhaler. Improvements on health literacy for asthma management and treatment were articulated as a priority from and for whānau [14, 45, 47, 48]. Verbal‐only explanations were common, and many clinicians admitted they struggled to gauge caregiver understanding. As one nurse reflected, “it's hard to tell how much they really understand*…* sometimes you can tell they're just nodding”^14(p.21)^. This quote illustrates the difficulty health professionals face in assessing asthma health literacy during routine consultations. Relying on non‐verbal cues such as nodding or body language is an unreliable proxy for comprehension, which may leave misunderstandings unrecognised and unaddressed. Verbal only teaching approaches combined with brief consultation times and poor continuity of care, limited opportunities to reinforce asthma knowledge or action plans over time [12, 14]. It was noted that parents, children and families/whanau would benefit from stepwise, hands‐on, and visual teaching tools that were developmentally appropriate and culturally relevant [45]. Jones et al. [14] found caregivers were more receptive to education that acknowledged their existing knowledge and engaged them as partners in care. Yet, many clinicians reported feeling underprepared to provide culturally tailored education, with one community nurse commenting, ‘it's not just about language—it's about knowing how to build trust and meet people where they are at’^14(p.24)^.

Building trusting, consistent relationships with healthcare professionals was identified as one way to improve health literacy/knowledge on asthma management and treatment [14, 47]. Of interest, Hebenton [47] revealed differing perspectives on ‘knowledge,’ with pharmacists viewing themselves as primary knowledge holders, while also recognizing that parents and children possess valuable insights. Conversely, McNamara et al. [45] advised health professionals to act on the assumption that there is a low level of asthma health literacy among child and whanau/family patients. This positioning holds that healthcare practitioners must specifically ask the child and whānau ‘what they understand, what they want to know*’*, as well as use simple language to explain asthma and ‘work with families to attain and maintain wellness, and not accept sickness as the norm*’* ^52(p.4)^. The ARFNZ [48] described the important role nurses play in delivering group‐based education sessions to whānau as part of or linked to existing programmes or activities.

Asthma education in school settings was also identified as an area of need. Families described a lack of understanding among teachers about asthma triggers and symptoms, as well as stigma associated with using inhalers in class and advocated for school based programmes to improve asthma awareness and reduce the stigma some children encounter when managing their asthma in public [12, 45, 48].

### 4.3 Children and Whanau/Family Experiences

Asthma care in children and young people under 18 years, is deeply embedded in family routines, relationships, responsibilities, self‐efficacy and systemic inequities in care. Across four studies, mothers in particular, were positioned as the ‘*primary knower’* of the child's condition, coordinating medications, recognising symptoms, and making decisions about escalation of care [39, 40, 47, 49]. Primary caregivers often described feeling an overwhelming responsibility for their child's care and management of their asthma [40, 47]. Parents reported feelings of stress and anxiety related to the speed at which symptoms could exacerbate. As one parent reflected,’*I do not want to be the sole knower of it all, but it's the way it's worked out’* ^46(p.13)^. Parents' confidence in handling the illness and having access to the necessary resources, such as knowledge and tools, or the sometimes unwanted responsibility of having to decide whether to manage at home or seek assistance may have contributed to this sense [40].

Poorly controlled asthma was found to negatively affect school attendance and performance [48, 50, 51]. Although no major differences in absenteeism between intervention and control groups were found, academic performance, particularly in reading, was affected [50]. Pattemore, Liberty and Reid [50] found children with asthma tended to be behind in reading, with those suffering from persistent asthma more likely to perform worse in word and story reading compared to their peers. Wiechern [51] found asthmatic children demonstrated slower breathing and longer pauses when reading difficult material, suggesting that asthma severity impacted cognitive tasks. School and community based asthma assessment and education programmes including sustainable funding from the government have been reported to positively impact children and whanau/families with the timing of these programmes key to achieve desired outcomes [48].

This review further confirmed the systemic inequities in asthma care, with Māori and Pacific children consistently experiencing poorer outcomes across multiple indicators [2, 40, 42, 46, 52]. Māori tamariki were less likely to receive inhaled corticosteroids, more likely to rely on oral steroids or nebulisers during acute episodes [14], and have higher rates of asthma‐related hospitalisation, longer stays [52], and readmission than non‐Māori children [2, 42, 46] These patterns reflect deeper structural inequities in health service delivery, access, and cultural safety.

### 4.4 Culture and Beliefs

Cultural beliefs further shaped how asthma was perceived and managed. In some families/whānau, preventer inhalers were viewed as unnecessary, particularly if the child appeared well most of the time. ‘Some members of his whānau were initially opposed to the use of a preventer … believing it could lead to weakness,’ one nurse reported [48, p. 22].

In the Jones and Ingham study, clinicians frequently acknowledged their discomfort or lack of preparation in providing culturally safe care [14]. They highlighted a need for better training, more time to build trust, and greater partnership with Māori health providers. One general practitioner noted, ‘they [kaupapa Māori services] do it better—because they take time, and it's not just medical*’* [14, p. 25]. Despite guidelines recommending culturally responsive approaches and the use of multilingual resources, uptake remains inconsistent across services [45].

Other studies pointed to the specific challenges faced by immigrant families. Sudarsan, Hoare, Sheridan and Roberts [49] described a ‘*tug‐of‐war*’ for Indian children navigating asthma, caught between biomedical advice and family‐held beliefs. Children reported being discouraged from using inhalers, fearing both side effects and social stigma. ‘Doctors say use it [preventer], but mum says it's not good,’ an eight‐year‐old participant shared [49, p. 4017].

The misalignment between parents beliefs and children's prescribed asthma care plan was also reported in the Trnka [39] study with some parents describing themselves as irresponsible for not following medical instructions. This suggests that their beliefs around asthma impacted on parental actions. These same parents did not always see asthma as a chronic condition, but instead as something that ‘fluctuated based on symptoms*’* ^47(p.555)^. This belief affected how they used medication and whether they perceived their children as ‘currently having asthma*’* ^47(p.555)^. Some parents believed their child would use their medication (steroid inhaler) unnecessarily and worried about side effects [40, 49]; these belief systems therefore impacted them encouraging their child to take their inhaled steroid medications.

The ARFNZ report [12] illustrated how concerns of parents about their children's asthma differed from adults' concerns about their own asthma. Concerns included access to health care with 65% being worried about getting an appointment when they needed one and 38% expressing concern about their child's ability to participate in sport or exercise [12].

## 5 Discussion

The purpose of this study was to identify and map existing literature on asthma care and management for children and young people aged under 18 years in Aotearoa New Zealand. Despite the presence of national clinical guidelines and well‐established evidence‐based approaches to asthma management, the findings reveal persistent challenges and opportunities in four key domains: medications and adherence, education and health literacy, children and whānau experiences, and cultural beliefs and attitudes. Across these domains, the review highlights a consistent pattern of inequities in asthma outcomes for Māori and Pacific children, variable implementation of preventative medication strategies, and ongoing reliance on verbal‐only and generalised education approaches that often fail to meet the specific needs of children and their families. The evidence reviewed points to a system that continues to privilege biomedical models over culturally grounded, family/whānau‐centred care, and the urgent need for policy and practice reform that centres on the lived realities and preferences of children and their caregivers.

Despite clear clinical guidance on the importance of regular preventer medication, this review found adherence to asthma treatment among children in Aotearoa New Zealand remains suboptimal, with significant variation by ethnicity and household factors [14, 37, 38]. Chan, Stewart, Foster, Mitchell, Camargo and Harrison [37] reported a median adherence rate of just 30%, with Māori children showing the lowest adherence of all groups—a pattern consistent with broader systemic inequities in access and support [2, 12, 14, 43, 46, 48, 52]. While technological interventions such as audiovisual electronic monitoring devices and reminder systems [36] show promise in improving adherence, their impact may be limited without addressing the underlying inequities in access, cultural safety, and continuity of care that disproportionately affect Māori and Pacific children [19, 53].

This review also highlighted the continued overreliance on short‐acting β2‐agonists (SABAs) and underuse of inhaled corticosteroids (ICS), despite longstanding evidence that this pattern is linked to poorer outcomes and increased risk of asthma‐related morbidity [14, 42, 45, 46]. Māori children were disproportionately prescribed SABA medications yet consistently received a lower proportion of corticosteroid prescriptions compared to non‐Māori [43, 46]. These findings raise concerns about treatment equity, the adequacy of asthma reviews in primary care, and whether current prescribing practices align with national guideline recommendations [45]. Addressing this issue requires not only better medication monitoring and follow‐up, but also a shift toward family/whānau‐centred prescribing and education practices that actively engage families in treatment planning and decision‐making [54].

Articles in this review revealed that caregivers often lacked a full understanding of asthma triggers, disease progression, and the rationale behind treatment plans, even when they were proficient in administering medications [12, 14, 40, 47]. One study in the review reported parent's self‐efficacy in managing asthma was found to be tied to parents' confidence and having access to resources that enhance knowledge [40]. This is consistent with other studies that identified parental self‐efficacy increased over time with their own asthma management successes and from observing the experiences of others rather than from formal instruction [55, 56, 57]. In addition, Brown, Gallagher, Fowler and Wales [56] found self‐efficacy was significantly higher for predictable, routine asthma tasks (such as giving medications or attending appointments) and lower for complex, infrequent tasks such as managing acute asthma exacerbations. This aligns with findings from our review, where parents reported struggling with timely escalation of care and management of exacerbations, often experiencing heightened anxiety and reduced confidence when symptoms worsened rapidly [40, 47]. Brown, Gallagher, Fowler and Wales [56] also highlighted when children grow older and begin to share responsibility for asthma care, parental self‐efficacy decreased, pointing to the need for ongoing support strategies with a focus on health literacy that evolves as the child develops [58].

The quality of the educational relationship with staff was also a central concern as family's/whānau consistently expressed a preference for stepwise, hands‐on guidance provided within trusted relationships, whereas clinicians often described time constraints and uncertainty about how to assess caregiver understanding [14]. These findings suggest that asthma education should not be viewed as a one‐off intervention but rather as an iterative, developmentally responsive process embedded within long‐term partnerships. This perspective is supported by international studies demonstrating that sustained, developmentally tailored asthma education programs particularly those involving community partnerships lead to improved asthma control and self‐management skills in children [59, 60]. Quaranta, Geoghegan, Gutierrez, Kim and Stala [60] evaluated a community university partnership delivering asthma education through schools and found significant improvements in children's asthma self‐efficacy and control. Furthermore, a systematic review of school‐based self‐management interventions emphasized the importance of parental involvement and age‐appropriate education, noting that programs with these components were more successful in improving health outcomes among primary school children [59].

The lack of asthma awareness in school environments and the persistence of stigma around inhaler use reported in this review, point to missed opportunities for reinforcing asthma literacy outside of clinical settings [12, 49]. Enhancing asthma education requires not only improved tools and cultural responsiveness [61], but also structural/system changes to enable clinicians to spend adequate time with families and collaborate more effectively with schools and community services [53, 62].

Whilst asthma care in children is factored in routines and becomes a collective effort of family and whanau, several studies have shown mothers are predominantly the caregivers of asthma patients [39, 40, 47, 49]. Similarly, de Araújo Gueiros Lira, Pontes da Silva and Sarinho [63] reports asthma care and management usually sits with the mother, as mother's have a strong influence on the physical, mental, and psychological well‐being of their children. Pars, Soyer and Şekerel [64], therefore highlights the need for healthcare providers to support parents more effectively, in particular mothers, thereby helping improve patient outcomes. Asthma Action Plans are one useful day‐to‐day asthma self‐management tool that can guide and support parents when a child is unwell [55].

In our review, children with asthma demonstrated poorer reading performance than their peers without asthma, with more severe asthma linked to greater cognitive disruption during learning tasks. This was similarly reported by Koinis‐Mitchell, Kopel, Farrow, McQuaid and Nassau [65] who concluded asthma was linked to poorer academic outcomes across multiple indicators, with some children facing heightened risk of underachievement due to the combined impact of health and social factors. Other studies reported severity of the child's asthma impacted on school attendance which in turn affected the child's reading performance [65, 66, 67]. Although we acknowledge the possibility this review did not capture all the literature pertaining to the research question, the existing literature has shown limited studies on asthma interventions or management programmes undertaken in schools within Aotearoa New Zealand. This highlights a critical gap and signals the need for further research to evaluate the role and effectiveness of school‐based interventions in supporting asthma management for children.

The findings in this review have reported Māori and Pasifika populations experience a greater burden of poorer health outcomes in asthma. Asthma management is not only a biomedical challenge but a socio‐cultural one, shaped by caregivers' worldviews, collective experiences, and the health system's responsiveness to diversity. International studies increasingly recognise how culture and health beliefs influence adherence and engagement with care among communities from racial and ethnically diverse backgrounds [68, 69, 70, 71, 72]. In one study, Chinese and Punjabi communities were concerned with the stigma that came with using inhalers in public due to the belief that asthma was a communicable disease that affects the lungs, like tuberculosis [68]. Such practices may be related to negative experiences, limited understanding or lack of sufficient education on what asthma is and its treatment and management from physicians to racial and ethnic minorities posed by implicit biases regarding cultural differences or intercultural miscommunication [68, 69, 70, 71, 72].

Concerns of asthma treatment and beliefs should not be overlooked. These beliefs are not necessarily irrational but reflect different understandings of wellness, strength, and risk that are not always recognised within mainstream asthma care models [72]. Clinicians need to acknowledge these cultural beliefs and understand the roots of such beliefs so that asthma self‐management can be discussed in terms that caregivers and wider family/whanau can understand [73]. Such practices will allow a more realistic representation and rigorous insights into making the doctor‐patient communication process more culturally competent. On a practical level, accounting for indigenous knowledge in tailoring asthma education materials and mainstream asthma models are more likely to build trust and potentially enhance acceptance of the intervention, improve asthma control and confidence in self‐managing asthma [74].

## 6 Clinical Relevance

The findings of this review have several implications for daily practice among clinicians working with children and adolescents with asthma in Aotearoa New Zealand. The evidence on poor medication adherence and over‐reliance on short‐acting β₂‐agonists points to the importance of regular medication reviews and proactive follow‐up, particularly for Māori and Pacific children who remain disproportionately affected. Clinicians should routinely check inhaler technique, reinforce the role of preventer medications at every opportunity, and ensure that every child has an up‐to‐date, appropriate asthma action plan shared with schools and the family.

Second, the review highlights the limitations of verbal‐only education. Clinicians can improve caregiver and child understanding by using iterative hands‐on teaching strategies, pictorial resources, and opportunities for demonstration and feedback. Nurses in the community and general practice setting are often well placed to provide this education. In addition, service providers who are focused on asthma education (such as Asthma New Zealand could be better utilised and supported across more communities in NZ).

Third, family and whānau experiences point to the need for clinicians to recognise the emotional and logistical burden of asthma care, especially on mothers as primary caregivers. Integrating family/whānau‐centred approaches, such as shared decision‐making and involving multiple caregivers, may help distribute responsibility and strengthen adherence. Finally, awareness of cultural beliefs and stigma around asthma management is essential. Clinicians should sensitively explore family perspectives, address misconceptions, and, where appropriate, collaborate with Māori and Pacific health providers to deliver care that is culturally grounded and thus more likely to be accepted. This scoping review provides the first comprehensive synthesis of asthma care and management literature focused specifically on children and young people under 18 years in Aotearoa New Zealand. A key strength of the review lies in its incorporation of both peer‐reviewed studies and grey literature, as well as national surveillance and guideline documents to ensure a broad understanding of current practice and policy contexts. However, the review also has several limitations. While every effort was made to identify relevant literature, studies capturing the lived experiences of tamariki with asthma appear underrepresented. In particular, there was a noticeable absence of children and young people's voices, with most studies privileging adult or caregiver perspectives. Additionally, this review did not assess the effectiveness of specific interventions or clinical outcomes, as its purpose was to map existing knowledge rather than evaluate impact. These limitations point to areas for future research, particularly qualitative inquiry centred on child and young people's perspectives and evaluations of culturally responsive, family/whānau‐centred interventions.

## 7 Conclusion

This scoping review synthesised a decade of research on asthma care and management for children and young people under 18 years in Aotearoa New Zealand, highlighting key themes related to medication adherence, health literacy, lived experiences, and cultural beliefs. Despite comprehensive national guidelines, asthma management remains inconsistent, particularly for Māori and Pacific children who experience disproportionately poorer outcomes. The review points to an urgent need for sustained, culturally responsive, and developmentally tailored interventions embedded within family, whānau and community contexts. Ongoing research must prioritise equity, elevate children and young people's voices, and support integrated models of care across health and education sectors to advance asthma outcomes for all children in Aotearoa, New Zealand.

## Author Contributions

JB and MF conceptualized the review. JB, MF, and BK‐N developed the methodology and search strategy. MF and BK‐N conducted the screening and data charting. JB led the synthesis and data analysis. JB wrote the original draft, and all authors contributed to reviewing and editing the manuscript. All authors read and approved the final manuscript.

## Ethics Statement

Ethical approval was not required for this scoping review as it involved analysis of publicly available literature and did not involve human participants or primary data collection.

## Conflict of Interest

The author(s) declared the following potential conflicts of interest with respect to the research, authorship, and/or publication of this article: there are no conflicts of interest to report.

## Data Availability

The data that support the findings of this study are openly available in CINAHL at https://research‐ebsco‐com.ezproxy.aut.ac.nz/c/cz6jai/search/advanced/filters?auth‐callid=3687fa50‐cb27‐4eca‐964d‐3f1e65ed2a1b&autocorrect=y&defaultdb=ccm.
